# A proteomics–metabolomics approach indicates changes in hypothalamic glutamate–GABA metabolism of adult female rats submitted to intrauterine growth restriction

**DOI:** 10.1007/s00394-018-1851-6

**Published:** 2018-11-07

**Authors:** Amanda P. Pedroso, Ana P. S. Dornellas, Adriana P. de Souza, Josias F. Pagotto, Lila M. Oyama, Cláudia M. O. Nascimento, Jelena Klawitter, Uwe Christians, Alexandre K. Tashima, Eliane Beraldi Ribeiro

**Affiliations:** 1 Departamento de Fisiologia, Universidade Federal de São Paulo, Escola Paulista de Medicina, Rua Botucatu 862, Vila Clementino, São Paulo, SP 04023-062 Brazil; 2 Departamento de Bioquímica, Universidade Federal de São Paulo, Escola Paulista de Medicina, São Paulo, SP Brazil; 3 iC42 Clinical Research and Development, Department of Anesthesiology, University of Colorado Denver, Anschutz Medical Campus, Aurora, CO USA

**Keywords:** Hypothalamus, Undernutrition, Pregnancy, Low birth weight, Obesity, Metabolic programming

## Abstract

**Purpose:**

Intrauterine growth restriction (IUGR) has been shown to induce the programming of metabolic disturbances and obesity, associated with hypothalamic derangements. The present study aimed at investigating the effects of IUGR on the protein and metabolite profiles of the hypothalamus of adult female rats.

**Methods:**

Wistar rats were mated and either had ad libitum access to food (control group) or received only 50% of the control intake (restricted group) during the whole pregnancy. Both groups ate ad libitum throughout lactation. At 4 months of age, the control and restricted female offspring was euthanized for blood and tissues collection. The hypothalami were processed for data independent acquisition mass spectrometry-based proteomics or targeted mass spectrometry-based metabolomics.

**Results:**

The adult females submitted to IUGR showed increased glycemia and body adiposity, with normal body weight and food intake. IUGR modulated significantly 28 hypothalamic proteins and 7 hypothalamic metabolites. The effects of IUGR on hypothalamic proteins and metabolites included downregulation of glutamine synthetase, glutamate decarboxylase, glutamate dehydrogenase, isocitrate dehydrogenase, α-ketoglutarate, and up-regulation of NADH dehydrogenase and phosphoenolpyruvate. Integrated pathway analysis indicated that IUGR affected GABAergic synapse, glutamate metabolism, and TCA cycle, highly interconnected pathways whose derangement has potentially multiple consequences.

**Conclusion:**

The present findings suggested that the effects of IUGR on GABA/glutamate–glutamine cycle may be involved in the programming of obesity and hyperglycemia in female rats.

**Electronic supplementary material:**

The online version of this article (10.1007/s00394-018-1851-6) contains supplementary material, which is available to authorized users.

## Introduction

The influence of low birth weight due to intrauterine growth restriction (IUGR) on the susceptibility of developing metabolic disorders later in life has been documented in humans [1–6]. The thrifty phenotype hypothesis proposes that malnutrition in early life can result in long-lasting alterations in structure and function of fetal tissues that are associated with the development of type 2 diabetes and metabolic syndrome at adulthood [7, 8]. Studies in rodent IUGR models have associated maternal caloric or protein restriction with hyperphagia and obesity in the adult offspring [9, 10].

The hypothalamus plays a crucial role in the maintenance of energy homeostasis and IUGR-induced hypothalamic disorders have been linked to obesity. A low protein diet throughout pregnancy and lactation induced morphological alterations in hypothalamic nuclei and high levels of hypothalamic neuropeptide Y (NPY) at weaning [11, 12]. Decreased expression of proopiomelanocortin mRNA levels in the hypothalamus of neonate rats whose dams were subjected to 50% caloric restriction has also been described [13].

The effects of metabolic programming due to intrauterine malnutrition have been shown to be gender specific [14–16]. We have previously shown that the IUGR-evoked alterations in the adult adipose tissue proteome were concordant with established obesity in female rats, while the males showed a metabolic status favoring later obesity development [17]. Our laboratory has also reported a more pronounced impairment of hypothalamic insulin action in female than in male rats submitted to IUGR [18].

We have recently shown deleterious consequences of IUGR in the hypothalamus of adult male rats. Impairment of glucose metabolism, respiratory chain and glutathione metabolism were observed, indicating alterations in mechanisms relevant to energy metabolism and redox homeostasis in this brain region at adulthood [19].

The current investigation, therefore, aimed at evaluating the long-term effects of IUGR, induced by calorie restriction, on the hypothalamus of female rats. Using a combined proteomics–metabolomics approach, we were able to identify potential pathways affected by undernutrition during pregnancy. The present results indicate that IUGR modified mechanisms related to GABAergic synapse, glutamate metabolism and TCA cycle in the hypothalamus of adult female rats.

## Materials and methods

### Animals

This study was approved by the Research Ethics Committee of the Universidade Federal de São Paulo—UNIFESP. Experimental procedures were conducted as described previously [19]. Briefly, pregnant 3-month-old female *Wistar* rats (*Rattus norvegicus*) were randomly assigned to be a control (fed ad libitum) or a restricted dam (fed 50% of control intake) throughout pregnancy. They were fed balanced chow (Nuvilab CR-1, Nuvital Nutrientes SA, Colombo, PR, Brazil) and kept under controlled light (12 h light:12 h dark cycle, lights on at 6 am) and temperature (22 ± 1 °C) conditions and had free access to water throughout the experimental period.

After parturition, the offspring were weighed and litter size was adjusted to eight (four males and four females) per dam. Dams from both groups received food *ad libitum* during lactation. From weaning up to 4 months of age, female offspring was fed ad libitum. Food intake and body weight were evaluated once a week in the course of this period.

### Sample collection

At 4 months of age, the rats were euthanized after 8 h of fasting; the hypothalamus was rapidly dissected and immediately frozen in liquid nitrogen. White fat depots (retroperitoneal, mesenteric and gonadal) were dissected and weighed. Trunk blood was collected and centrifuged at 1125×*g* for 20 min at 4 °C. Serum aliquots were stored at − 80 °C until further analysis.

### Serum and tissue measurements

Glucose and triglycerides levels were determined using commercially available kits (Labtest Diagnóstica, Lagoa Santa, MG, Brazil). Serum leptin, insulin and adiponectin levels as well as hypothalamic levels of TNF-α, IL-6 and IL-10 were measured by Elisa (Millipore, Bedford, MA, USA). Briefly, the hypothalami (*n* = 6–8) were homogenized in 1 mL of chilled extraction buffer containing 100 mM Trizma Base pH 7.5, 10 mM EDTA, 100 mM NaF, 10 mM Na_4_P_2_O_7_, 10 mM Na_3_VO_4_, 2 mM PMSF, and 0.1 mg/ml aprotinin. After centrifugation at 20,800×*g* for 40 min at 4 °C, the supernatants were recovered and aliquots of 100 µL were used for protein assessment according to the manufacturer´s recommendations.

### Proteomic analysis

#### Sample preparation

Extraction of hypothalamic proteins was performed as described previously [19]. Briefly, the whole frozen hypothalamus was homogenized in lysis buffer 8 M urea, 75 mM NaCl, 1 M Tris and complete Mini Protease Inhibitor Cocktail Tablets (Roche Diagnostics, Indianapolis, IN, USA) and protein concentration was determined (2-D Quant Kit, GE Healthcare, Waukesha, WI, USA). One milligram of sample protein was subjected to dialysis (Amicon Ultra-4 Centrifugal 3000 NMWL filter, Merck Millipore) against 50 mM ammonium bicarbonate. Then, 200 µg of concentrated protein were heated for 15 min at 80 °C in the presence of 25 µL of 0.2% RapiGest SF (Waters, Milford, MA, USA), reduced with 100 mM DTT, alkylated with 300 mM iodoacetamide and digested with trypsin (Promega, Fitchburg, WI, USA). The samples were then acidified with 5% trifluoroacetic acid, centrifuged and the supernatants transferred to the vials for MS analysis (Waters).

#### Mass spectrometry and data analysis

Hypothalamic samples (*n* = 4) were analyzed in triplicates on a nanoAcquity UPLC system coupled to a Synapt G2 HDMS Q-TOF mass spectrometer (Waters). Samples (5 µL) were loaded onto a trap column (nanoAquity C18 trap column Symmetry 180 µm × 20 mm, Waters) and transferred by an elution gradient (phase B gradient from 7 to 35% for 92 min at a 275 nL/min) through the analytical column (nanoAcquity C18 BEH 75 µm × 150 mm, 1.7 mm, Waters). The mobile phase A was 0.1% formic acid in water and the mobile phase B was 0.1% formic acid in acetonitrile. Data were acquired in data-independent mode (HDMS^E^), switching from low (4 eV) to high (ramped from 19 to 45 eV) collision energy. For external calibration, Glu-fibrinopeptide B solution (500 fmol/mL in 50% acetonitrile, 0.1 formic acid; Waters) was infused at 500 nL/min every 30 s using a nanoLockSpray apparatus.

Data were processed using the ProteinLynx Global Server software version 3.0.1 (Waters) with database search against *Rattus norvegicus* sequences in the UniProtKB/Swiss-Prot database (http://www.uniprot.org, including 9485 entries). The search parameters included automatic precursor and fragment mass tolerance, two missed cleavage sites allowed for trypsin digestion, cysteine carbamidomethylation as fixed modification and methionine oxidation, N-terminal acetylation, glutamine and asparagine deamidation as variable modifications. Additionally, the protein identification criteria included a minimum of one fragment ion per peptide, five fragment ions per protein and two peptides per protein. The false discovery identification rate was set at 4%. Label-free quantitative assessments based on peptide intensities were performed by integrating the intensities of the three most intense peptides of each identified protein [20]. Results were exported into Excel files and normalization was performed using the sum of protein intensities. For relative quantitation, only proteins identified in at least two technical replicates of at least three biological replicates were considered. Additionally, proteins not detected in any of the 12 replicates of one group (indicating that the intensities were below the detection limit), but identified in at least 4 replicates in the other group were listed and included in the pathway analysis.

### Metabolomic analysis

#### Sample preparation

All procedures were carried out based on a previously described LC–MS/MS platform [21]. After whole hypothalamus homogenization (*n* = 8) in 500 µL of 80% precooled methanol, samples were incubated for 4 h at − 0 °C, centrifuged at 14,000×*g* for 10 min at 4 °C and the supernatant was collected. The remained pellet was mixed with 400 µL of 80% (v/v) methanol, followed by 30 min incubation at − 80 °C and a second centrifugation at the same conditions as described above. The combined supernatants were dried in a SpeedVac concentrator centrifuge (Thermo Fisher, Waltham, MA, USA). Extraction of serum metabolites was performed by mixing 200 µL of samples (*n* = 8) with 800 µL of precooled methanol. After incubation for 6 h at − 80 °C, samples were centrifuged at 14,000×*g* for 10 min at 4 °C and the supernatants dried in a SpeedVac.

#### Mass spectrometry and data analysis

Dried samples were reconstituted in 50% (v/v) acetonitrile. Methionine-d3 was used as an internal standard. The chromatographic separation was performed in an Agilent 1100 series HPLC system (Agilent Technologies, Santa Clara, CA, USA). Metabolites were separated using a 3.0 × 150 mm 3 µm Luna HILIC column (Phenomenex, Torrance, CA, USA). The mobile phase A was 5% acetonitrile, 20 mM ammonium hydroxide, 20 mM ammonium acetate (pH 9.0) and the mobile phase B was 100% acetonitrile. The gradient was 85–30% of phase B during 3 min and to 2% during 9 min. This condition was maintained for 3 min and increased back to 85% for 1 min. The column was reconditioned for 7 min before the next injection. The HPLC system was coupled to an ABSciex 5500 hybrid triple quadrupole mass spectrometer (Sciex, Framingham, MA, USA) operating with an electrospray ionization source (ESI) in positive/negative switch mode. The Q1 > Q3 transitions for positive/negative ion switching for the targeted metabolites were analogue to the analytical platform previously described [21, 22].

Raw data were extracted using the MarkerView v1.2.1 software (Sciex) to the following parameters: gaussian smooth width of 2 points, peak splitting factor of 5 points, one peak limited per chromatogram, a minimum intensity of 1000 cps, a minimum signal/noise ratio of 5 and a minimum peak width of 5 points. Retention time correction was performed based on methionine-d3. Only metabolites showing a single peak in the chromatogram were included in the analysis and the result tables were exported into Excel files. Normalization of hypothalamic metabolites was performed using the sum of intensities of all quantified metabolites while normalization of serum metabolites used methionine-d3 intensity.

### Statistical analysis

Statistical analysis was performed in Statistica 12 Software (StatSoft, Tulsa, OK, USA). Body weight, white fat depots mass, food intake and serum and hypothalamic parameters are expressed as mean and standard error. Significance of the differences between the restricted and the control group was determined using Student’s *t* test. Statistical significance was set at *p* < 0.05.

### Interaction network and pathway analysis

Interaction network of all proteins and metabolites affected by IUGR was generated using STITCH (http://stitch.embl.de/) [23], with confidence score fixed at 0.4 and no additional proteins allowed.

Pathway over-representation analysis was performed using the web server IMPaLA (http://impala.molgen.mpg.de/) [24]. Multiple testing correction was performed with the false discovery rate method (FDR, Benjamini–Hochberg) and significance was set to *q* ≤ 0.01.

## Results

### Body and fat depots mass, food intake and serum parameters

The body weight of the restricted group was significantly lower than that of the control group from birth (Table 1) until the eleventh week of age (data not shown). However, at 4 months of age, the body weight of the restricted group was no longer significantly decreased (Table 1).

**Table 1:** Body mass, food intake and fat depots mass of the control and restricted groups

	Control	Restricted
Body weight at birth (g)	5.93 ± 0.10 (12)	4.99 ± 0.11*** (12)
Body weight at weaning (g)	84.77 ± 1.45 (12)	71.75 ± 2.17*** (12)
Body weight at 4 months (g)	241.40 ± 3.92 (12)	231.37 ± 3.37 (12)
Food intake at 4 months (g/100 g)	6.08 ± 0.15 (12)	6.13 ± 0.17 (9)
Retroperitoneal fat depot (g/100 g)	0.84 ± 0.04 (12)	0.94 ± 0.05 (12)
Gonadal fat depot (g/100 g)	2.46 ± 0.16 (12)	3.01 ± 0.18** (12)
Mesenteric fat depot (g/100 g)	1.04 ± 0.04 (12)	1.25 ± 0.06** (12)
Fat depots sum (g/100 g)	4.35 ± 0.22 (12)	5.20 ± 0.22* (12)

Values are means ± SEM; (number of animals)

*p < 0.05; **p < 0.01; ***p < 0.001

Since weaning, there were no differences in food intake between the control and the restricted groups, except at the sixth and fourteenth weeks, when the restricted offspring ate 7% more and 5% less, respectively, than the controls (data not shown). As shown in Table 1, food intake was similar between the groups at 4 months of age.

The mass of gonadal and mesenteric white adipose tissues, as well as the sum of fat depots (retroperitoneal, gonadal and mesenteric), were significantly higher in the restricted than in the control group (Table 1).

A significant increase of glucose levels was detected in the restricted group while no significant differences were detected in serum insulin, triglycerides, leptin and adiponectin levels. Hypothalamic cytokine levels were similar between the groups (Table 2).

**Table 2:** Serum and hypothalamic parameters of the control and restricted groups at 4 months of age

	Control	Restricted
Serum glucose (mg/dL)	105.97 ± 3.86 (12)	122.75 ± 4.90* (12)
Serum insulin (ng/mL)	0.44 ± 0.05 (7)	0.50 ± 0.09 (7)
Serum triglycerides (mg/dL)	44.97 ± 3.10 (9)	49.68 ± 5.46 (9)
Serum leptin (ng/mL)	3.84 ± 0.58 (10)	3.90 ± 0.37 (10)
Serum adiponectin (µg/mL)	23.84 ± 3.37 (12)	23.77 ± 5.20 (9)
Hypothalamic TNF-α (pg/mL)	45.96 ± 6.13 (8)	49.69 ± 4.15 (8)
Hypothalamic IL-6 (pg/mL)	79.81 ± 4.79 (6)	82.63 ± 6.34 (7)
Hypothalamic IL-10 (pg/mL)	134.63 ± 30.64 (8)	114.40 ± 17.73 (8)

Values are means ± SEM; (number of animals)

*p < 0.05

### Proteomics

The female rat hypothalamus dataset comprised of 1475 proteins, each one identified by at least 2 peptides, with false discovery rate set to 4%. After application of the inclusion criteria for quantification (presence in at least 2 technical replicates and 3 biological replicates), 419 proteins were compared between the groups.

Table 3 shows the hypothalamic proteins significantly affected by IUGR. Ten proteins were down-regulated while 2 proteins were up-regulated in the hypothalamus of the restricted group. Additionally, 13 proteins were identified only in the control group, while 3 proteins were identified only in the restricted group. Glutamine synthetase, glutamate dehydrogenase 1 (mitochondrial) and glutamate decarboxylase 2, proteins which participate in glutamate–glutamine metabolism, were all down-regulated in the restricted group. Table S1 (supplementary material) details MS information on the proteins affected by IUGR.

**Table 3:** Hypothalamic proteins significantly affected by intrauterine growth restriction

UniProt ID	Gene name	Protein	Fold change (restricted/control)	*p* value
Down-regulated by IUGR				
Q05683	Gad2	Glutamate decarboxylase 2	0.49	0.0063
P30009	Marcks	Myristoylated alanine-rich C-kinase substrate	0.62	0.0422
Q9Z269	Vapb	Vesicle-associated membrane protein-associated protein B	0.62	0.0159
Q6Q0N1	Cndp2	Cytosolic non-specific dipeptidase	0.68	0.0321
O35567	Atic	Bifunctional purine biosynthesis protein PURH	0.72	0.0072
P85969	Napb	Beta-soluble NSF attachment protein	0.74	0.0367
P09606	Glul	Glutamine synthetase	0.77	0.0368
P10860	Glud1	Glutamate dehydrogenase 1, mitochondrial	0.77	0.0494
P70580	Pgrmc1	Membrane-associated progesterone receptor component 1	0.80	0.0244
Q62952	Dpysl3	Dihydropyrimidinase-related protein 3	0.92	0.0206
O08875	Dclk1	Serine/threonine-protein kinase DCLK1	Not detected in the restricted group^a^	
P05696	Prkca	Protein kinase C alpha type		
P16446	Pitpna	Phosphatidylinositol transfer protein alpha isoform		
P18666	Myl12b	Myosin regulatory light chain 12B		
P41562	Idh1	Isocitrate dehydrogenase [NADP] cytoplasmic		
P60522	Gabarapl2	Gamma-aminobutyric acid receptor-associated protein-like 2		
P62198	Psmc5	26S protease regulatory subunit 8		
P62870	Elob/Tceb2	Elongin-B		
Q4V8E4	Cfap36	Cilia- and flagella-associated protein 36		
Q62915	Cask	Peripheral plasma membrane protein CASK		
Q66HR2	Mapre1	Microtubule-associated protein RP/EB family member 1		
Q75Q39	Tomm70	Mitochondrial import receptor subunit TOM70		
Q9R0I8	Pip4k2a	Phosphatidylinositol 5-phosphate 4-kinase type-2 alpha		
Up-regulated by IUGR				
P19234	Ndufv2	NADH dehydrogenase [ubiquinone] flavoprotein 2, mitochondrial	1.24	0.0386
P27682	Scg5	Neuroendocrine protein 7B2	1.17	0.0275
O08775	Kdr	Vascular endothelial growth factor receptor 2	Not detected in the control group^b^	
P02688-2	Mbp	Myelin basic protein (Isoform 2)		
P51146	Rab4b	Ras-related protein Rab-4B		

*UniProt* universal protein resource

^a^Down-regulation by IUGR

^b^Up-regulation by IUGR

### Metabolomics

The analysis of the hypothalamic samples included 131 different metabolites that met the inclusion criterion (presence of a single chromatographic peak). Multiple reaction monitoring (MRM) transitions are shown in Table 4. The restricted group had decreased levels of 5 metabolites and increased levels of 2 metabolites in the hypothalamus when compared to the controls (Table 4). Among the hypothalamic metabolites altered by IUGR, 2 are involved in the TCA cycle (α-ketoglutarate and phosphoenolpyruvate) and 2 take part in purine metabolism (deoxyguanosine and guanosine).

**Table 4:** Hypothalamic metabolites significantly affected by intrauterine growth restriction

KEGG entry	Metabolite	Single reaction monitoring Q1/Q3	Fold change (restricted/control)	*p* value
Down-regulated by IUGR				
C03626	ADMA (*N,N*-dimethylarginine)	203.2/46.2	0.76	0.045
C00330	Deoxyguanosine	268.1/152.0	0.78	0.032
C00029	UDP-d-glucose	565.0/323.0	0.78	0.036
C00065	Serine	106.0/60.0	0.82	0.013
C00026	α-Ketoglutarate	145.0/101.0	0.87	0.049
Up-regulated by IUGR				
C00387	Guanosine	284.1/135.0	1.31	0.020
C00074	Phosphoenolpyruvate	167.0/79.0	1.31	0.049

*KEGG* Kyoto Encyclopedia of Genes and Genomes

### Interaction network and pathway analysis

The analysis showed significant enrichment of the interaction network of hypothalamic proteins and metabolites affected by IUGR (*p* = 0.00149). IUGR induced hypothalamic alterations were related to GABAergic synapse, alanine, aspartate and glutamate metabolism, TCA cycle, arginine biosynthesis, glyoxylate and dicarboxylate metabolism and HIF-1 signaling pathway (Table 5).

**Table 5:** Integrated pathway analysis of hypothalamic proteins and metabolites significantly affected by intrauterine growth restriction

Pathway	Protein	Metabolite	*p* value	*q* value
GABAergic synapse	Glutamine synthetase (↓)	α-Ketoglutarate (↓)	0.0000057	0.0012
	Gamma-aminobutyric acid receptor-associated protein-like 2 (↓)			
	Protein kinase C alpha type (↓)			
	Glutamate decarboxylase 2 (↓)			
Alanine, aspartate and glutamate metabolism	Glutamine synthetase (↓)	α-Ketoglutarate (↓)	0.0000272	0.0037
	Glutamate dehydrogenase 1, mitochondrial (↓)			
	Glutamate decarboxylase 2 (↓)			
Citrate cycle (TCA cycle)	Isocitrate dehydrogenase [NADP] cytoplasmic (↓)	Phosphoenolpyruvate (↑)	0.000286	0.0111
		α-Ketoglutarate (↓)		
Arginine biosynthesis	Glutamine synthetase (↓)	α-Ketoglutarate (↓)	0.000326	0.0121
	Glutamate dehydrogenase 1, mitochondrial (↓)			
Glyoxylate and dicarboxylate metabolism	Glutamine synthetase (↓)	Serine (↓)	0.00203	0.0369
		α-Ketoglutarate (↓)		
HIF-1 signaling pathway	Protein kinase C alpha type (↓)	α-Ketoglutarate (↓)	0.0037	0.0432
	Elongin-B (↓)			

Arrows indicates protein or metabolite down (↓) or up-regulation (↑)

*p* value for pathway over-representation analysis; *q* value: corrected *p* values false discovery rate, Benjamini–Hochberg

## Discussion

The existence of a relationship between low birth weight and the development of metabolic disturbances in adulthood has been established. In agreement with ours [17, 19, 25] and other reports [26, 27] in both genders, here we observed that 50% calorie restriction throughout pregnancy induced low birth weight of the female offspring. At adulthood, these restricted females had normal body weight but increased body fat, in accordance with earlier data [9, 28]. As adults, the restricted females were hyperglycemic with normal levels of insulin, indicating alteration of the glucose-insulin metabolism, a feature also previously reported as an IUGR effect [29–31].

Since hypothalamic inflammation has been implicated as a relevant contributor to the development of obesity [32, 33], we investigated the inflammatory status of the hypothalamus and found no significant effects of IUGR on TNF-α, IL-6 and IL-10 tissue levels. Unlike these present cytokines results obtained in basal conditions, it has been previously demonstrated that IUGR increased the hypothalamic response of IL-1β, TNF-α and IL-6 mRNA to systemic lipopolysaccharide (LPS) challenge [34].

The hypothalamic proteome and metabolome profile indicated that IUGR affected GABAergic synapse, glutamate metabolism, and TCA cycle, all pathways that are highly interconnected.

The amination of α-ketoglutarate, a TCA intermediate, yields glutamate. Conversely, glutamate may generate α-ketoglutarate through the glutamate dehydrogenase reaction. In neurons and astrocytes, these reactions occur in a dynamic equilibrium, aimed at both assuring adequate levels of neurotransmitters and preventing impairment of the oxidative capacity of the cell and ATP depletion [35, 36].

IUGR down-regulated the levels of α-ketoglutarate, isocitrate dehydrogenase (the enzyme catalyzing the production of α-ketoglutarate in the TCA cycle), and glutamate dehydrogenase, indicating impaired α-ketoglutarate metabolism. In contrast, IUGR upregulated the levels of phosphoenolpyruvate (the precursor of pyruvate in glycolysis), possibly as a compensatory mechanism. Within neurons, anaplerotic carboxylation of pyruvate to yield oxaloacetate has been shown to compensate for the loss of α-ketoglutarate intrinsic to glutamatergic and GABAergic neurotransmission [37]. In the rodent cerebral cortex, a coupling between the rate of glucose oxidation and the flux of the GABA/glutamate–glutamine cycle has been demonstrated [38].

In the present study, the hypothalamic levels of the enzymes glutamine synthetase, and glutamate decarboxylase were down-regulated by IUGR. The excitatory neurotransmitter glutamate released by neurons undergoes uptake and conversion to glutamine by glutamine synthetase in astrocytes [35, 38]. Glutamine is released and transported back to neurons, where it acts as a precursor for the synthesis of glutamate by phosphate-activated glutaminase. Glutamate is decarboxylated to yield the inhibitory neurotransmitter γ-aminobutyric acid (GABA), a reaction catalyzed by glutamate decarboxylase in GABAergic neurons [39].

The current observation that IUGR affected GABAergic synapses and glutamate metabolism are in agreement with several lines of evidence in rodents. An association of IUGR, induced by either food deprivation or ligature of uterine vessels, and disturbances in the GABAergic system has been previously suggested by the finding of reduced glutamate decarboxylase activity in several brain regions [40, 41]. A proteomic approach has shown altered glutamate metabolism in the adult frontal cortex of a maternal low protein diet rat model [42]. We have previously shown that both hypothalamic and serum levels of glutamate were decreased in male rats submitted to IUGR [19]. Obesity and type 2 diabetes have also been associated with GABA/glutamate–glutamine cycle deregulation [43].

The roles of hypothalamic glutamate and GABA signaling in the control of energy balance have deserved attention. Studies in rodents have shown feeding stimulation by injection of glutamate agonists in the lateral hypothalamic area [44, 45] and impaired glucose homeostasis by inactivation of vesicular glutamate transporter in the ventromedial hypothalamus [46]. In vitro and in vivo studies have shown that hyperglycemia induced impairment of glutamate metabolism in neurons and astrocytes [47, 48].

The involvement of hypothalamic GABA signaling in the control of systemic glucose concentration has been previously demonstrated, as blockade of GABA receptors in the paraventricular nucleus increased plasma glucose concentrations [49]. Moreover, disruption of GABA release from a specific subset of non-AgRP non-POMC neurons of the arcuate nucleus of mice induced obesity through decreasing energy expenditure [50]. The present decrease in the hypothalamic expression of glutamate decarboxylase, reported as an indicator of GABA release [51], may be involved in the high plasma glucose and adiposity observed in female rats exposed to IUGR.

The relationship between disturbances of insulin and leptin signaling and disorders of the GABAergic synapse and glutamate metabolism in the hypothalamus of IUGR animals remains to be determined. Hypothalamic impairment of insulin and leptin actions due to IUGR has been previously shown by us and others [18, 52, 53]. Inactivation of insulin receptors in GABAergic neurons led to increased body weight and adiposity in female mice [54]. Moreover, leptin acts on GABAergic neurons to produce its anorexigenic effect [55] and also modulates glutamate uptake in hypothalamic astrocytes [56]. It is interesting to point out that here we observed decreased levels of UDP-glucose, which is a precursor of membrane glycosphingolipids. It has been demonstrated that glycosphingolipids-derived gangliosides take part in central leptin signaling and its neuronal deletion induces obesity in mice [57].

It has been reported that the changes induced by IUGR are gender-dependent [15–18, 58, 59]. We have recently used the proteomics/metabolomics approach to examine the consequences of IUGR in the hypothalamus of adult male rats. The data indicated increased glucose levels and enhanced flux through the hexosamine pathway, along with effects on energy metabolism and redox homeostasis [19]. The findings presented herein strengthen the gender dimorphism in the response to IUGR. Interestingly, glutamate and GABA neuronal systems are sexually distinct in several hypothalamic nuclei, namely the medial preoptic area, the ventromedial nucleus and lateral area [60].

It has been shown in sheep that caloric restriction during pregnancy caused maternal hypoglycemia [61]. The ventromedial hypothalamic nucleus, whose GABAergic network is not fully developed before birth [62], has glucose-sensing neurons, which have been shown to be blunted by hypoglycemic episodes [63] and modulated by 17β-estradiol [64]. One might infer that glucose-sensing neurons in the ventromedial nucleus may be involved in the distinct responses to IUGR between genders.

In conclusion, the present results indicate that IUGR induced changes in hypothalamic GABA/glutamate–glutamine cycle of female rats, a derangement with potentially multiple consequences. The present data suggested obesity and hyperglycemia as results of the effect of IUGR on this pivotal neurotransmitters pathway.

## Electronic supplementary material

Below is the link to the electronic supplementary material.

Supplementary material 1 (PDF 51 KB)

## References

[1] Ravelli GP, Stein ZA, Susser MW (1976). Obesity in young men after famine exposure in utero and early infancy. N Engl J Med.

[2] Hales CN, Barker DJ, Clark PM, Cox LJ, Fall C, Osmond C, Winter PD (1991). Fetal and infant growth and impaired glucose tolerance at age 64. BMJ.

[3] Law CM, de Swiet M, Osmond C, Favers PM, Barker DJ, Cruddas AM, Fall CH (1993). Initiation of hypertension in utero and its amplification throughout life. BMJ.

[4] Curhan GC, Chertow GM, Willet WC, Spiegelman D, Colditz GA, Manson JE, Speizer FE, Stampfer MJ (1996). Birth weight and adult hypertension and obesity in women. Circulation.

[5] Curhan GC, Willet WC, Rimm EB, Spiegleman D, Ascherio AL, Stampfer MJ (1996). Birth weight and adult hypertension, diabetes mellitus, and obesity in US men. Circulation.

[6] Roseboom T, de Rooij S, Painter R (2006). The Dutch famine and its long term consequences for adult health. Early Hum Dev.

[7] Hales CN, Barker DJP (1992). Type 2 (non-insulin-dependent) diabetes mellitus: the thrifty phenotype hypothesis. Diabetologia.

[8] Hales C, Barker D (2001). The thrifty phenotype hypothesis. Br Med Bull.

[9] Vickers M, Breier B, Cutfield WS, Hofman PL, Gluckman PD (2000). Fetal origins of hyperphagia, obesity, and hypertension and postnatal amplification by hypercaloric nutrition. Am J Physiol Endocrinol Metab.

[10] Desai M, Gayle D, Han G, Ross MG (2007). Programmed hyperphagia due to reduced anorexigenic mechanisms in intrauterine growth-restricted offspring. Reprod Sci.

[11] Plagemann A, Harder T, Rake A, Melchior K, Rohde W, Dörner G (2000). Hypothalamic nuclei are malformed in weanling offspring of low protein malnourished rat dams. J Nutr.

[12] Plagemann A, Waas T, Harder T, Rittel F, Ziska T, Rohde W (2000). Hypothalamic neuropeptide Y levels in weanling offspring of low-protein malnourished mother rats. Neuropeptides.

[13] Delahaye F, Breton C, Risold PY, Enache M, Dutriez-Casteloot I, Laborie C, Lesage J, Vieau D (2008). Maternal undernutrition drastically reduces postnatal leptin surge and affects the development of arcuate nucleus proopiomelanocortin neurons in neonatal male rat pups. Endocrinology.

[14] Ravelli ACJ, van der Meulen JHP, Osmond C, Barker DJP, Blecker OP (1999). Obesity at the age of 50 y in men and women exposed to famine prenatally. Am J Clin Nutr.

[15] Palou M, Priego T, Sánchez J, Palou A, Picó C (2010). Sexual dimorphism in the lasting effects of moderate caloric restriction during gestation on energy homeostasis in rats is related with fetal programming of insulin and leptin resistance. Nutr Metab.

[16] Picó C, Palou M, Priego T, Sánchez J, Palou A (2012). Metabolic programming of obesity by energy restriction during the perinatal period: different outcomes depending on gender and period, type and severity of restriction. Front Physiol.

[17] Souza AP, Pedroso AP, Watanabe RL, Dornellas AP, Boldarine VT, Laure HJ, Nascimento CM, Oyama LM, Rosa JC, Ribeiro EB (2015). Gender-specific effects of intrauterine growth restriction on the adipose tissue of adult rats: a proteomic approach. Proteome Sci.

[18] Sardinha FLC, Telles MM, Albuquerque KT, Oyama LM, Guimarães PA, Santos OF, Ribeiro EB (2006). Gender difference in the effect of intrauterine malnutrition on the central anorexigenic action of insulin in adult rats. Nutrition.

[19] Pedroso AP, Souza AP, Dornellas APS, Oyama LM, Nascimento CM, Santos GM, Rosa JC, Bertolla RP, Klawitter J, Christians U, Tashima AK, Ribeiro EB (2017). Intrauterine growth restriction programs the hypothalamus of adult male rats: integrated analysis of proteomic and metabolomics data. J Proteome Res.

[20] Silva JC, Gorenstein MV, Li GZ, Vissers JPC, Geromanos SJ (2006). Absolute quantification of proteins by LCMSE: a virtue of parallel MS acquisition. Mol Cell Proteom.

[21] Yuan M, Breitkopf SB, Yang X, Asara JM (2012). A positive/negative ion-switching, targeted mass spectrometry-based metabolomics platform for bodily fluids, cells, and fresh and fixed tissue. Nat Protoc.

[22] Checkley W, Deza MP, Klawitter J, Romero KM, Klawitter J, Pollard SL, Wise RA, Christians U, Hansel NN (2016). Identifying biomarkers for asthma diagnosis using targeted metabolomics approaches. Respir Med.

[23] Szklarczyk D, Santos A, von Mering C, Jensen LJ, Bork P, Kuhn M (2016). STITCH 5: augmenting protein–chemical interaction networks with tissue and affinity data. Nucleic Acids Res.

[24] Kamburov A, Cavill R, Ebbels TMD, Herwig R, Keun HC (2011). Integrated pathway-level analysis of transcriptomics and metabolomics data with IMPaLA. Bioinformatics.

[25] Pôrto LC, Sardinha FL, Telles MM, Guimarães RB, Albuquerque KT, Andrade IS, Oyama LM, Nascimento CM, Santos OF, Ribeiro EB (2009). Impairment of the serotonergic control of feeding in adult female rats exposed to intra-uterine malnutrition. Br J Nutr.

[26] Landgraf MA, Landgraf RG, Silva RC, Semedo P, Câmara NOS, Fortes ZB (2012). Intrauterine undernourishment alters TH1/TH2 cytokine balance and attenuates lung allergic inflammation in *Wistar* rats. Cell Physiol Biochem.

[27] Tungalagsuvd A, Matsuzaki T, Iwasa T, Munkhzaya M, Yiliyasi M, Kawami T, Kato T, Kuwahara A, Irahara M (2016). The expression of orexigenic and anorexigenic factors in middle-aged female rats that had been subjected to prenatal undernutrition. Int J Dev Neurosc.

[28] Guan H, Arany E, van Beek JP, Chamson-Reig A, Thyssen S, Hill DJ, Yang K (2005). Adipose tissue gene expression profiling reveals distinct molecular pathways that define visceral adiposity in offspring of maternal protein-restricted rats. Am J Physiol Endocrinol Metab.

[29] Jones RH, Ozanne SE (2009). Fetal programming of glucose-insulin metabolism. Mol Cell Endocrinol.

[30] Martin-Gronert MS, Ozanne SE (2012). Metabolic programming of insulin action and secretion. Diabetes Obes Metab.

[31] Garg M, Thamotharan M, Dai Y, Lagishetty V, Matveyenko AV, Lee WN, Devaskar SU (2013). Glucose intolerance and lipid metabolic adaptations in response to intrauterine and postnatal calorie restriction in male adult rats. Endocrinology.

[32] de Souza CT, Araujo EP, Bordin S, Ashimine R, Zollner RL, Boschero AC, Saad MJ, Velloso LA (2005). Consumption of a fat-rich diet activates a proinflammatory response and induces insulin resistance in the hypothalamus. Endocrinology.

[33] Cesar HC, Pisani LP (2017). Fatty-acid-mediated hypothalami inflammation and epigenetic programming. J Nutr Biochem.

[34] Iwasa T, Matsuzaki T, Tungalagsuvd A, Munkhzaya M, Kuwahara A, Yasui T, Irahara M (2015). Prenatal undernutrition increases the febrile response to lipopolysaccharides in adulthood in male rats. Int J Dev Neurosc.

[35] El Hage M, Masson J, Conjard-Duplany A, Ferrier B, Baverel G, Martin G (2012). Brain slices from glutaminase-deficient mice metabolize less glutamine: a cellular metabolomics study with carbon 13 NMR. J Cereb Blood Flow Metab.

[36] Sonnewald U (2014). Glutamate synthesis has to be matched by its degradation—where do all the carbons go?. J Neurochem.

[37] Hassel B (2000). Carboxylation and anaplerosis in neurons and glia. Mol Neurobiol.

[38] Behar KL, Rothman DL (2001). In vivo nuclear magnetic resonance studies of glutamate-gamma-aminobutyric acid cycling in rodent and human cortex: the central role of glutamine. J Nutr.

[39] Schousboe A, Waagepetersen HS (2006). Glial modulation of GABAergic and glutamatergic neurotransmission. Curr Top Med Chem.

[40] Patel AJ, del Vecchio M, Atkinson DJ (1978). Effect of undernutrition on the regional development of transmitter enzymes: glutamate decarboxylase and choline acetyltransferase. Dev Neurosc.

[41] Chanez C, Rabin O, Heroux M, Giguere JF (1993). Cerebral amino acid changes in an animal model of intrauterine growth retardation. Metab Brain Dis.

[42] Guest PC, Urday S, Ma D, Stelzhammer V, Harris LW, Amess B, Pietsch S, Ohein C, Ozanne SE, Bahn S (2012). Proteomic analysis of the maternal protein restriction rat model for schizophrenia: identification of translational changes in hormonal signaling pathways and glutamate neurotransmission. Proteomics.

[43] Sickmann RM, Waagepetersen HS, Schousboe A, Benie AJ, Bouman SD (2010). Obesity and type 2 diabetes in rats are associated with altered brain glycogen and amino-acid homeostasis. J Cereb Blood Flow Metab.

[44] Stanley BG, Urstadt KR, Charles JR, Kee T (2011). Glutamate and GABA in lateral hypothalamic mechanisms controlling food intake. Physiol Behav.

[45] Charles JR, Duva MA, Ramirez GJ, Lara RL, Yang CR, Stanley BG (2014). Activation of lateral hypothalamic mGlu1 and mGlu5 receptors elicits feeding in rats. Neuropharmacology.

[46] Tong Q, Ye C, McCrimmon RJ, Dhilon H, Choi B, Kramer MD, Yu J, Yang Z, Christiansen LM, Lee CE, Choi CS, Zigman JM, Shulman GI, Sherwin RS, Elmquist JK, Lowell BB (2007). Synaptic glutamate release by ventromedial hypothalamic neurons is part of the neurocircuitry that prevents hypoglycemia. Cell Metab.

[47] Rivera-Aponte DE, Méndez-Gonzáles MP, Rivera-Pagán AF, Kucheryavykh YV, Kucheryavykh LY, Skatchkov SN, Eaton MJ (2015). Hyperglycemia reduces functional expression of astrocytic Kir4.1 channels and glial glutamate uptake. Neuroscience.

[48] Zheng Y, Yang Y, Dong B, Zheng H, Lin X, Du Y, Li X, Zhao L, Gao H (2016). Metabonomic profiles delineate potential role of glutamate-glutamine cycle in db/db mice with diabetes-associated cognitive decline. Mol Brain.

[49] Kalsbeek A, La Fleus S, Van Heijningen C, Buijs RM (2004). Suprachiasmatic GABAergic inputs to the paraventricular nucleus control plasma glucose concentrations in the rat via sympathetic innervation of the liver. J Neurosci.

[50] Kong D, Tong Q, Ye C, Koda S, Fuller PM, Krashes MJ, Vong L, Ray RS, Olson DP, Lowell BB (2012). GABAergic RIP-Cre neurons in the arcuate nucleus selectively regulate energy expenditure. Cell.

[51] Dicken MS, Hughes AR, Hentges ST (2015). Gad1 mRNA as a reliable indicator of altered GABA release from orexigenic neurons in the hypothalamus. Eur J Neurosci.

[52] Yura S, Itoh H, Sagawa N, Yamamoto H, Masuzaki H, Nakao K, Kawamura M, Takemura M, Kakui K, Ogawa Y, Fujii S (2005). Role of premature leptin surge in obesity resulting from intrauterine undernutrition. Cell Metab.

[53] Coupé B, Grit J, Hulin P, Randuineau G, Parnet P (2012). Postnatal growth after intrauterine growth restriction alters central leptin signal and energy homeostasis. PLoS One.

[54] Evans MC, Rizwan MZ, Anderson GM (2014). Insulin action on GABA neurons is a critical regulator of energy balance but not fertility in mice. Endocrinology.

[55] Vong L, Ye C, Yang Z, Choi B, Chua SJ, Lowell BB (2011). Leptin action on GABAergic neurons prevents obesity and reduces inhibitory tone to POMC neurons. Neuron.

[56] Fuente-Martín E, García-Cáceres C, Granado M, de Ceballos ML, Sánchez-Garrido M, Sarman B, Liu ZW, Dietrich MO, Tena-Sempere M, Argente-Arizón P, Díaz F, Argente J, Horvath TL, Chowen J (2012). Leptin regulates glutamate and glucose transporters in hypothalami astrocytes. J Clin Investig.

[57] Nordström V, Willershäuser M, Herzer S, Rozman J, von Halbach B, Meldner O, Rothermel S, Kaden U, Roth S, Waldeck FC, Gretz C, de Angelis N, Draguhn MH, Klingenspor A, Gröne M, Jannemann HJ (2013). Neuronal expression of glucosylceramide synthase in central nervous system regulates body weight and energy homeostasis. PLoS Biol.

[58] García-Contreras D, Valent D, Vázquez-Gómez M, Arroyo L, Isabel B, Astiz S, Bassols A, Gonzalez-Bulnes A (2017). Fetal growth-retardation and brain-sparing by malnutrition are associated to changes in neurotransmitters profile. Int J Dev Neurosci.

[59] Ramírez-López MT, Vázquez M, Lomazzo E, Hofmann C, Blanco RN, Alén F, Antón M, Decara J, Arco R, Orio L, Suárez J, Lutz B, Gómez de Heras R, Bindila L, de RodríguezFonseca F (2017). A moderate diet restriction during pregnancy alters the levels of the endocannabinoids and endocannabinoid-related lipids in the hypothalamus, hippocampus and olfactory bulb of the rat offspring in a sex-specific manner. PLoS One.

[60] Frankfurt M, Fuchs E, Wuttke W (1984). Sex differences in γ-aminobutyric acid and glutamate concentration in discrete rat brain nuclei. Neurosci Lett.

[61] Dellschaft NS, Alexandre-Gouabau MC, Gardner DS, Antignac JP, Keisler DH, Budge H, Symonds ME, Sebert SP (2015). Effect of pre- and postnatal growth and post-weaning activity on glucose metabolism in the offspring. J Endocrinol.

[62] Kobayashi M, Shimizu-Okabe C, Kim J, Kobayashi S, Matsushita M, Masuzaki H, Takayama C (2017). Embryonic development of GABAergic terminals in the mouse hypothalamic nuclei involved in feeding behavior. Neurosci Res.

[63] Song Z, Routh VH (2006). Recurrent hypoglycemia reduces the glucose sensitivity of glucose-inhibited neurons in the ventromedial hypothalamus nucleus. Am J Physiol Regul Integr Comp Physiol.

[64] Santiago AM, Clegg DJ, Routh VH (2016). Estrogens modulate ventrolateral ventromedial hypothalamic glucose-inhibited neurons. Mol Metab.

