# Supplementary material for: A proteomics–metabolomics approach indicates changes in hypothalamic glutamate–GABA metabolism of adult female rats submitted to intrauterine growth restriction
Source: Eur J Nutr. 2018 Nov 7;58(8):3059–68. doi: 10.1007/s00394-018-1851-6 (PMC6842332; doi:10.1007/s00394-018-1851-6)
Supplement: Supplementary file 1 — Supplementary material 1 (PDF 51 KB) [file 394_2018_1851_MOESM1_ESM.pdf]

European Journal of Nutrition

A proteomics-metabolomics approach indicates changes in hypothalamic glutamate-GABA metabolism of adult female rats submitted to intrauterine growth restriction

Amanda P. Pedroso[1], Ana P. S. Dornellas[1], Adriana P. de Souza[1], Josias F. Pagotto[2], Lila M. Oyama[1], Cláudia M. O. Nascimento[1], Jelena Klawitter[3], Uwe Christians[3], Alexandre K. Tashima[2], Eliane B. Ribeiro[1*]

[1]Universidade Federal de São Paulo, Escola Paulista de Medicina, Departamento de Fisiologia, São Paulo, SP, Brazil.

[2]Universidade Federal de São Paulo, Escola Paulista de Medicina, Departamento de Bioquímica, São Paulo, SP, Brazil.

[3]iC42 Clinical Research and Development, Department of Anesthesiology, University of Colorado Denver, Anschutz Medical Campus, Aurora, CO, USA.

***Corresponding author**

Eliane Beraldi Ribeiro

Universidade Federal de São Paulo, Departamento de Fisiologia.

Rua Botucatu 862

Vila Clementino, 04023-062, São Paulo, SP, Brazil.

E-mail: eliane.beraldi@gmail.com

Phone/Fax: 55 11 5576-4765

Supplementary Table S1: Mass spectrometry information of the proteins significantly affected by intrauterine growth restriction in the adult female rat hypothalamus

UniProt ID	Protein Score			False Positive Rate (%)			Matched Peptides			Sequence Coverage (%)		
	Min	Mean	Max	Min	Mean	Max	Min	Mean	Max	Min	Mean	Max
Q05683	301.61	1018.05	2328.58	0.00	0.03	0.27	7	12	20	12.48	29.33	55.73
P30009	280.14	542.68	1338.87	0.00	0.11	0.58	4	7	10	14.24	29.69	40.78
Q9Z269	199.53	996.07	2346.66	0.00	0.05	0.71	3	7	10	14.40	40.68	67.49
Q6Q0N1	179.80	613.15	1435.27	0.00	0.23	1.84	7	12	18	13.26	35.03	62.95
O35567	160.75	360.23	983.07	0.00	0.45	2.03	6	14	21	9.12	27.68	38.18
P85969	1735.76	4977.41	10540.24	0.00	0.00	0.00	6	14	20	32.32	54.90	75.42
P09606	720.89	2598.49	4983.32	0.00	0.00	0.00	8	11	16	18.50	38.56	59.52
P10860	2782.06	5556.65	13023.67	0.00	0.00	0.00	28	32	38	51.61	62.34	72.22
P70580	1131.48	2304.16	5065.62	0.00	0.00	0.00	6	10	15	43.08	58.27	67.69
Q62952	580.53	1500.86	4659.94	0.00	0.00	0.00	11	17	25	33.51	42.19	56.34
O08875	165.54	379.41	893.73	0.00	0.50	1.37	4	5	9	9.70	17.41	32.10
P05696	114.35	232.64	331.15	0.23	1.35	3.64	6	12	15	4.46	15.90	28.42
P16446	149.83	292.78	602.51	0.14	0.72	2.30	8	11	15	24.72	36.62	46.13
P18666	358.15	547.04	778.20	0.00	0.10	0.35	5	8	11	32.56	41.98	54.65
P41562	230.71	338.03	444.22	0.00	0.35	0.75	7	11	15	17.39	26.73	35.99
P60522	435.32	649.85	803.79	0.00	0.00	0.00	2	4	5	17.09	25.93	36.75
P62198	384.49	505.40	756.37	0.00	0.03	0.13	9	11	16	25.37	28.42	31.53
P62870	331.91	613.70	892.64	0.00	0.09	0.43	4	5	7	26.27	53.05	83.90
Q4V8E4	181.15	265.24	350.68	0.00	0.68	1.53	4	7	9	8.16	21.11	48.98
Q62915	143.65	237.46	380.85	0.12	1.06	2.05	10	17	20	11.11	14.28	23.32
Q66HR2	255.05	444.29	777.50	0.00	0.26	0.46	5	6	7	20.52	29.03	38.06
Q75Q39	112.65	251.65	423.75	0.00	1.18	3.48	9	14	22	8.69	15.01	19.02
Q9R0I8	137.98	219.71	259.81	0.12	1.21	2.19	6	7	8	9.36	16.31	28.33
P19234	6669.66	10226.03	17460.82	0.00	0.00	0.00	5	8	10	29.44	41.97	57.66
P27682	571.82	1497.89	4009.49	0.00	0.00	0.00	2	5	8	18.57	46.94	62.38
O08775	165.55	226.99	326.44	0.95	1.82	3.19	14	20	29	7.15	9.26	13.18
P02688-2	26914.30	33091.01	36623.99	0.00	0.00	0.00	8	10	12	43.20	54.84	60.95
P51146	1313.86	3585.33	5456.31	0.00	0.00	0.00	3	5	7	19.25	30.42	36.62
